# Supplementary material for: Aligning large language models and geometric deep models for protein representation
Source: Patterns (N Y). 2025 Apr 11;6(5):101227. doi: 10.1016/j.patter.2025.101227 (PMC12142629; doi:10.1016/j.patter.2025.101227)
Supplement: Document S1. Figures S1–S6 and Notes S1–S11 [file mmc1.pdf]

Patterns, Volume *6*

Supplemental information

Aligning large language models and geometric deep models for protein representation

Dong Shu, Bingbing Duan, Kai Guo, Kaixiong Zhou, Jiliang Tang, and Mengnan Du

Supplemental Notes
Aligning Large Language Models and Geometric Deep Models for Protein Representation

Dong Shu[1], Bingbing Duan[2], Kai Guo[3], Kaixiong Zhou[4], Jiliang Tang[3], and Mengnan Du*[5]

[1]Northwestern University, Computer Science Dept, Evanston, IL, 60201, USA
[2]University of Pittsburgh, Biological Sciences Dept, Pittsburgh, PA, 15260, USA
[3]Michigan State University, Computer Science Dept, East Lansing, MI, 48824, USA
[4]North Carolina State University, Electrical and Computer Engineering Dept, Raleigh, NC, 27695, USA
[5]New Jersey Institute of Technology, Data Science Dept, Newark, NJ, 07102, USA
[*]Correspondence: mengnan.du@njit.edu
[1]Lead: dongshu2024@u.northwestern.edu

Supplemental Notes: Table of Contents

1 Dataset Statistic

2 Prompt Used in FASTA Preprocess

3 Protein Examples

4 GDM Representation Details

5 Popular and Rare Protein

6 GearNet Training

7 Number of Projection Head Layer

8 LLM Finetune Implementation Details

9 Protein-focused MLLMs Methodology

10 Reweighting Methodology

11 Retrieval Methodology.

Figure S1 Data Statistic.

Figure S2 Single-Layer to Multi-Layer Projection Head: Architecture and Results.

Figure S3 Architecture of Protein-Focused MLLMs and Their Generated Responses.

Figure S4 Popular Protein Examples.

Figure S5 Rare Protein Examples.

Figure S6 Example of Protein-focused MLLMs Generated Output for Protein ID 3I1A.

1 Dataset Statistic

Our dataset consists of 20,000 proteins, each with an associated FASTA file and PDB file. Detailed statistics for the dataset are presented in Figure S1, where each pie chart represents a different categorical breakdown of the same dataset. For the “Number of Chains” statistic, the chart shows the count of single-chain proteins versus multiple-chain proteins, with further subdivisions indicating the exact number of chains for multi-chain proteins. For the “Sequence Length” statistic, proteins are categorized by sequence length range. Each section of the pie chart is labeled with the relevant number of chains or sequence length range at the top, with the exact count and corresponding percentage displayed below each label.

2 Prompt Used in FASTA Preprocess

During the FASTA file preprocessing stage, we used GPT-4o to handle proteins with multiple chains. Below is the GPT prompt we employed for this task:

```
==== System Prompt ====
You are a biologist with expertise in protein sequence analysis.
Your task is to summarize complex protein sequence data into two or
three sentences that highlight key features such as molecute type,
chains, structural motifs, organism, etc.

==== User Query ====
Summarize the following protein knowledge, start with the sentence:
'The protein structure {protein_id} has a sequence length of:
{sequence_length} amino acids.'
Here is more information about {protein_id}:
{fasta_text}
```

3 Protein Examples

As shown in Figure S4 and S5, we listed several proteins and their corresponding description. The meaning of ‘popular’ and ‘rare’ protein is discussed in Research Question 3: Protein Perspective Analysis.

4 GDM Representation Details

4.1 GearNet

Protein Graph Construction: The structure of a protein is represented as a residue-level relational graph $G = (V, E, R)$, where V represents the set of nodes, E the set of edges, and R the set of edge types. Each node $v_i \in V$ corresponds to a residue in the protein, with its 3D coordinates $\mathbf{x}_i \in \mathbb{R}^3$, and each edge $e_{ij} \in E$ represents a relationship between residues based on either sequential proximity or spatial proximity. In particular, GearNet incorporates three types of edges:

- **Sequential edges:** connect residues within a distance of 2 in the sequence.

- **Radius edges:** connect residues whose C_α atoms are within a given radius in 3D space.
- **K-nearest neighbor edges:** connect each residue to its k-nearest neighbors in 3D space.

Each node v_i is initially represented by its residue type and spatial coordinates, while each edge e_{ij} is represented by its edge type (sequential or spatial) and spatial distance.

Relational Graph Convolution: GearNet applies relational message passing on the constructed protein graph, leveraging both node and edge features. The relational graph convolutional layer is defined as:

$$h_i^{(0)} = f_i, \quad u_i^{(l)} = \sigma\left(\text{BN}\left(\sum_{r\in R} W_r \sum_{j\in\mathcal{N}_r(i)} h_j^{(l-1)}\right)\right), \quad h_i^{(l)} = h_i^{(l-1)} + u_i^{(l)}$$

where f_i is the initial feature of node i, $\mathcal{N}_r(i)$ is the set of neighbors connected to node i via edges of type r, W_r is a learnable weight matrix for edge type r, and σ is a ReLU activation function. Batch normalization (BN) is applied for normalization across batches, and a residual connection is added from the previous layer to stabilize training. This relational message passing allows GearNet to effectively capture both sequential and spatial information in the protein structure, as different edge types are modeled with different convolutional kernels.

Edge Message Passing: In addition to node message passing, GearNet incorporates an edge message-passing mechanism to explicitly model interactions between edges. The model constructs an edge-level graph $G' = (V', E', R')$, where each node in G' corresponds to an edge in the original protein graph G. The edge message passing is defined as:

$$m_{(i,j,r)}^{(0)} = f_{(i,j,r)}, \quad m_{(i,j,r)}^{(l)} = \sigma\left(\text{BN}\left(\sum_{r'\in R'} W'_{r'} \sum_{(w,k,r')\in\mathcal{N}'_{r'}((i,j,r))} m_{(w,k,r')}^{(l-1)}\right)\right)$$

where $m_{(i,j,r)}^{(l)}$ represents the message for edge (i,j,r) at layer l, $\mathcal{N}'_{r'}((i,j,r))$ is the set of neighboring edges in G', and $W'_{r'}$ is a learnable weight matrix for edge type r'. The angular information between edges is used to determine edge types, with edges having smaller angles expected to interact more strongly. The output from the edge message passing is integrated into the node update step by modifying the aggregation function as follows:

$$u_i^{(l)} = \sigma\left(\text{BN}\left(\sum_{r\in R} W_r \sum_{j\in\mathcal{N}_r(i)} \left(h_j^{(l-1)} + \text{FC}(m_{(j,i,r)}^{(l)})\right)\right)\right)$$

where FC is a fully connected layer applied to the edge message.

Protein Representation: After multiple rounds of relational graph convolution and edge message passing, each residue v_i in the protein is represented by a feature vector $h_i^{(L)}$, where L is the number of layers in the model. In the original GearNet model, the hidden dimensions are set to hidden_dims $= [512, 512, 512, 512, 512, 512]$, meaning that each residue is represented by a feature vector of size 512 after each layer. To obtain the final protein representation, the features from all layers are concatenated:

$$h_{\text{protein}} = \left[h_i^{(1)}, h_i^{(2)}, \ldots, h_i^{(6)}\right] \in \mathbb{R}^{3072}$$

where the concatenation of six layers (each of dimension 512) results in a 3072-dimensional feature vector for each protein. The final protein representation has the shape $[1, 3072]$.

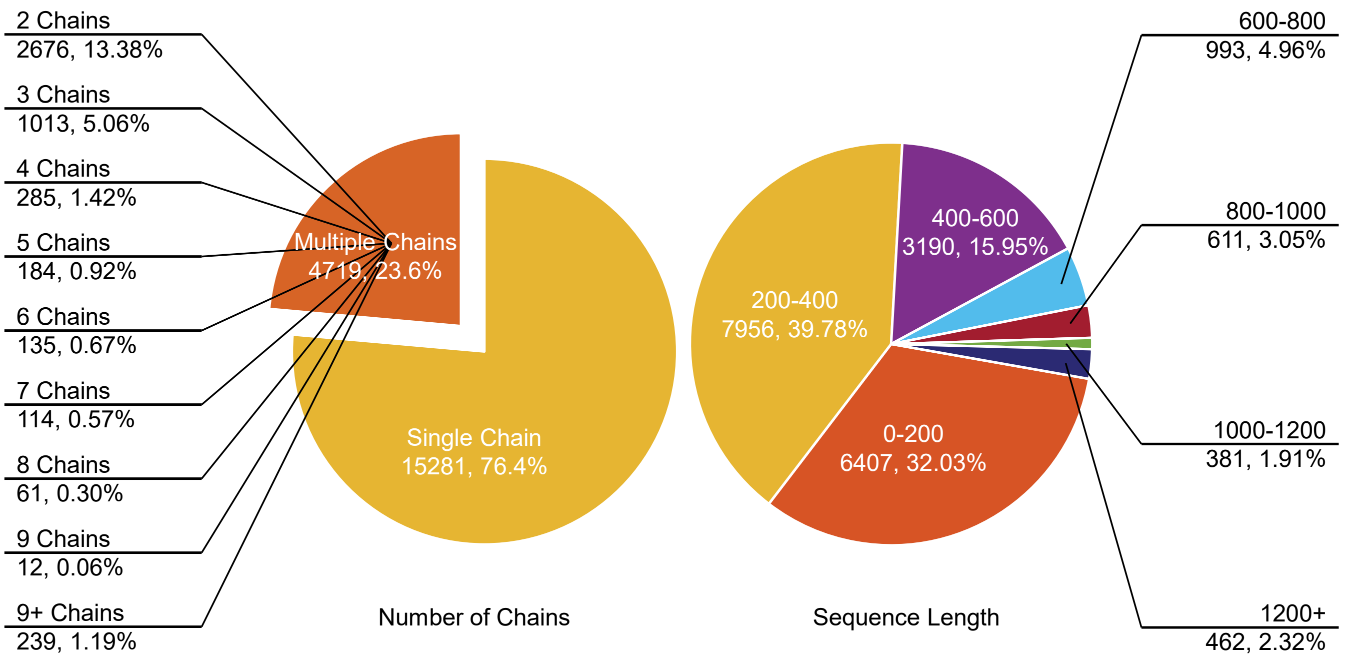

Figure S1: **Data Statistic.** The pie chart on the left categorizes proteins by the number of chains, while the pie chart on the right categorizes them by sequence length.

4.2 GVP

Graph Construction: Using the JSON input format, each protein's backbone structure is parsed to identify each residue and its corresponding coordinates for the N, C-alpha, C, and O atoms. This [num_residues x 4 x 3] nested list provides precise spatial positioning essential for structural insights. For each residue, a node $v_i \in V$ is created in the graph G, with scalar and vector features, and edges $e_{ij} \in E$ are created between nodes based on spatial proximity between residues. Scalar features $h_s \in \mathbb{R}^{n_s}$ include properties such as the amino acid type and backbone dihedral angles (e.g., ϕ, ψ, ω). Vector features $h_v \in \mathbb{R}^{n_v \times 3}$ encode geometric information, such as the direction of the residue's neighbors. These features are processed through a series of GVP layers, which operate on both scalar and vector features to propagate information through the graph.

Message Passing and Geometric Vector Perceptrons: The core of the GVP model is its message-passing architecture, which updates both scalar and vector features at each graph propagation step. For each node v_i, the message from a neighboring node v_j is computed using the scalar and vector features of both nodes and their connecting edge:

$$h_m^{(j\to i)} = \text{GVP}\left(\left[h_s^{(j)}, h_v^{(j)}, h_e^{(j\to i)}\right]\right)$$

where $h_s^{(j)}$ and $h_v^{(j)}$ are the scalar and vector features of node j, and $h_e^{(j\to i)}$ is the edge embedding, which includes both distance and direction information. The GVP function processes these features, maintaining rotation-equivariance for the vector features:

$$h_{v'}^{(i)} = \sigma\left(\sum_{j\in\mathcal{N}(i)} \mathbf{W}_v h_v^{(j)}\right), \quad h_{s'}^{(i)} = \sigma\left(\mathbf{W}_s h_s^{(i)} + \|\mathbf{W}_v h_v^{(j)}\|\right)$$

where $\mathbf{W}_v$ and $\mathbf{W}_s$ are learnable weight matrices for vector and scalar features, respectively, and σ is a non-linear activation function.

Protein Representation: After multiple rounds of message passing, each node v_i is associated with a scalar feature vector $h_s^{(i)} \in \mathbb{R}^{100}$ and a vector feature matrix $h_v^{(i)} \in \mathbb{R}^{16\times3}$. To construct the final protein representation, the scalar and vector features are combined. The vector features are reduced to scalars by taking their L2-norm:

$$h_v^{(i)} = \|\mathbf{h_v^{(i)}}\|_2$$

The final per-node embedding is then formed by concatenating the scalar and vector features:

$$h^{(i)} = \left[h_s^{(i)}, h_v^{(i)}\right] \in \mathbb{R}^{148}$$

where 148 is the dimension of the combined scalar and vector features (100 scalar channels and 3 vector channels of size 16, i.e., $3 \times 16 + 100 = 148$). Thus, the representation for the entire protein has the shape $[1, \text{node\_size}, 148]$, where $[\text{node\_size}]$ corresponds to the number of residues in the protein. Finally, to obtain a fixed-size representation for the entire protein, we apply average pooling across the node dimension:

$$h_{\text{protein}} = \frac{1}{\text{node\_size}} \sum_{i=1}^{\text{node\_size}} h^{(i)}$$

This results in a protein representation of shape $[1, 148]$, which will be stored and used later.

4.3 ScanNet

Parsing the PDB File: First, the PDB file is parsed to extract the protein's amino acid sequence and atomic point cloud. Each atom is represented as a triplet:

$$\{(\mathbf{x}_l, \text{id}_{\text{residue}_l}, \text{id}_{\text{atom}_l}) \mid l \in [1, N_{\text{atoms}}]\}$$

where $\mathbf{x}_l \in \mathbb{R}^3$ represents the atomic coordinates, and $\text{id}_{\text{residue}_l}$ and $\text{id}_{\text{atom}_l}$ represent the residue and atom IDs, respectively. Only heavy atoms belonging to classical residues are considered.

Local Frame Construction: Next, a local reference frame is defined for each heavy atom based on the molecular graph of the protein (i.e., nodes represent atoms and edges represent covalent bonds). For each atom, we select two neighboring atoms to construct a local coordinate system. The center is defined by the atom itself, and the orientation is determined by its neighbors using the following equations:

$$f_{l1} = \mathbf{x}_{\text{atom}_l}$$

$$f_{l4} = \frac{\mathbf{x}_{\text{neighbor}_3} - \mathbf{x}_{\text{atom}_l}}{\|\mathbf{x}_{\text{neighbor}_3} - \mathbf{x}_{\text{atom}_l}\|}$$

$$f_{l3} = \frac{f_{l4} \times (\mathbf{x}_{\text{neighbor}_2} - \mathbf{x}_{\text{atom}_l})}{\|f_{l4} \times (\mathbf{x}_{\text{neighbor}_2} - \mathbf{x}_{\text{atom}_l})\|}$$

$$f_{l2} = \frac{f_{l3} \times f_{l4}}{\|f_{l3} \times f_{l4}\|}$$

This frame is then used to transform atomic neighborhoods into a consistent local coordinate system.

Atomic and Amino Acid Pooling: ScanNet constructs atomic representations by extracting the local neighborhood of each atom and encoding it with spatio-chemical filters. These filters operate on the atomic coordinates and attributes (such as atom type) and produce an atomic-scale representation. This process is mathematically formalized using spatio-chemical Gaussian filters:

$$y_m = \text{ReLU}\left(\sum_{k,g,n} W_{mgn} G(\mu_g, \Sigma_g, x_k) a_k\right)$$

where $G(\mu_g, \Sigma_g, x_k)$ is the Gaussian kernel parameterized by mean μ_g and covariance Σ_g, applied to the local coordinates x_k of the neighborhood. The atomic-scale representations are then pooled at the amino acid level. The pooling step aggregates the atom-wise features into a single representation for each amino acid using a multi-head attention mechanism:

$$\mathbf{h}_{\text{AA}} = \sum_{i=1}^{N_{\text{atoms}}} \alpha_i \mathbf{h}_{\text{atom}_i}$$

where α_i are learned attention weights that determine the contribution of each atom to the amino acid representation.

Amino Acid Neighborhood Embedding and Protein Representation: For each amino acid, its local neighborhood is constructed based on the C_α atom, sidechain orientation, and backbone orientation. The same spatio-chemical filtering process is applied to obtain an amino acid-wise representation:

$$\mathbf{h}_{\text{AA}} = \text{ReLU}\left(\sum_{k,g,n} W_{mgn} G(\mu_g, \Sigma_g, x_k) a_k\right)$$

where $G(\mu_g, \Sigma_g, x_k)$ is the Gaussian kernel applied to the neighborhood of each amino acid. The amino acid representations are then aggregated to form a protein-level representation. This representation has the shape of $[1, \text{node\_size}, 128]$, where node_size corresponds to the number of amino acids in the protein, and each amino acid has a 128-dimensional latent feature vector. Then, we simply use the average pooling to reduce the node_size dimension, leaving us with $[1, 128]$ for each protein:

$$\mathbf{h}_{\text{protein}} = \frac{1}{\text{node\_size}} \sum_{i=1}^{\text{node\_size}} \mathbf{h}_{\text{AA}_i}$$

4.4 GAT

For a graph with N nodes, the input to the GAT layer is a set of node features $\mathbf{h} = \{\mathbf{h}_1, \mathbf{h}_2, \ldots, \mathbf{h}_N\}$, where $\mathbf{h}_i \in \mathbb{R}^F$ is the feature vector of node i, and F is the number of features associated with each atom. Each node is first transformed linearly using a shared learnable weight matrix $W \in \mathbb{R}^{F' \times F}$, yielding: $\mathbf{h}'_i = W\mathbf{h}_i$. Then, the attention mechanism computes attention coefficients for each pair of nodes i and j, where $j \in \mathcal{N}_i$ is a neighboring node of i. The attention coefficients e_{ij} are given by: $e_{ij} = \text{LeakyReLU}\left(\mathbf{a}^T \left[W\mathbf{h}_i \parallel W\mathbf{h}_j\right]\right)$, where $\mathbf{a} \in \mathbb{R}^{2F'}$ is a learnable weight vector, and $\parallel$ denotes concatenation. These attention coefficients are then normalized across all neighbors of node i using the softmax function: $\alpha_{ij} = \frac{\exp(e_{ij})}{\sum_{k \in \mathcal{N}_i} \exp(e_{ik})}$. The normalized attention coefficients are used to compute the updated feature representation of node i as a weighted sum of its neighbors' features: $\mathbf{h}''_i = \sigma\left(\sum_{j \in \mathcal{N}_i} \alpha_{ij} W\mathbf{h}_j\right)$ where σ is a non-linear activation function.

To stabilize the learning process, GAT employs multi-head attention, where K independent attention mechanisms compute the attention coefficients and aggregate the features. The outputs from the different attention heads are concatenated as: $\mathbf{h}_i^{\text{multi}} = \|_{k=1}^{K} \sigma\left(\sum_{j \in \mathcal{N}_i} \alpha_{ij}^k W^k \mathbf{h}_j\right)$. For the final prediction layer, the attention heads are averaged rather than concatenated: $\mathbf{h}_i^{\text{final}} = \sigma\left(\frac{1}{K}\sum_{k=1}^{K}\sum_{j \in \mathcal{N}_i} \alpha_{ij}^k W^k \mathbf{h}_j\right)$. In GAT architecture, they use $K = 8$ attention heads, with each head computing $F' = 8$ features, leading to a concatenated output of size 64 for each node, which results in protein's final representation size of [1, node_size, 64]. Then we simply perform average pooling to remove the 'node_size' dimension. This process yields the latent representation of the protein with size of [1, 64], which will be stored and used later.

5 Popular and Rare Protein

For the protein perspective analysis, we randomly selected 10 popular proteins and 10 rare proteins to study. The IDs of the popular proteins are: [4NWH, 2RAY, 4I8S, 3E3D, 4H8Y, 4H92, 1B1V, 3FE0, 3PYK, 3S9T], while the IDs of the rare proteins are: [3I1A, 4Q2G, 4S3K, 3GEU, 3OZQ, 3LOV, 1NVI, 3MGC, 4J77, 2GRM]. Details for these proteins are presented in Figure S4 and S5.

Before random selection, we first collected all proteins' molecule names and organisms. We then used regular expressions to clean the names, excluding specific terms like numbers within parentheses, and combined the molecule names and organisms into distinct category labels. Next, we mapped all the proteins to these categories and calculated the count for each. Proteins within categories with higher counts were considered as popular, while those in categories with lower counts (or a count of one) were considered rare. We then organized the proteins in ascending order from rarest to most popular and randomly selected 10 popular proteins from the top 100 most popular categories and 10 rare proteins from the top 100 rarest categories.

6 GearNet Training

To analyze whether the representation dimension size of the Geometric Deep Model (GDM) affects the alignment performance of the model pair, we trained GearNet using the Enzyme Commission dataset, following the tutorial steps from TorchDrug. We followed the original GearNet model configuration with an input dimension of 21, 7 node relations, 59 edge features, and 8 angle bins. The hidden layer size was adjusted to control the representation dimension, using the following configurations: {[64], [128], [256], [512], [512, 512], [512, 512, 512, 512, 512, 512]}. This resulted in six different dimension sizes: 64, 128, 256, 512, 1024, and 3072. We stopped at 3072, as it matches the dimension size of the original pretrained GearNet model. Batch normalization and a shortcut mechanism were applied, along with a sum-based readout layer for node feature aggregation. The model was trained using a binary cross-entropy loss function and optimized with the Adam optimizer. We evaluated performance using the AUPRC@micro and F1_max metrics. The training was conducted for 10 epochs with a batch size of 4.

7 Number of Projection Head Layer

To address Research Question 5, we investigate whether increasing the number of layers in the projection head improves alignment performance. As shown in Figure S2A, we only modify the number of linear layers in the GDM's projection head, while leaving the LLM's projection head

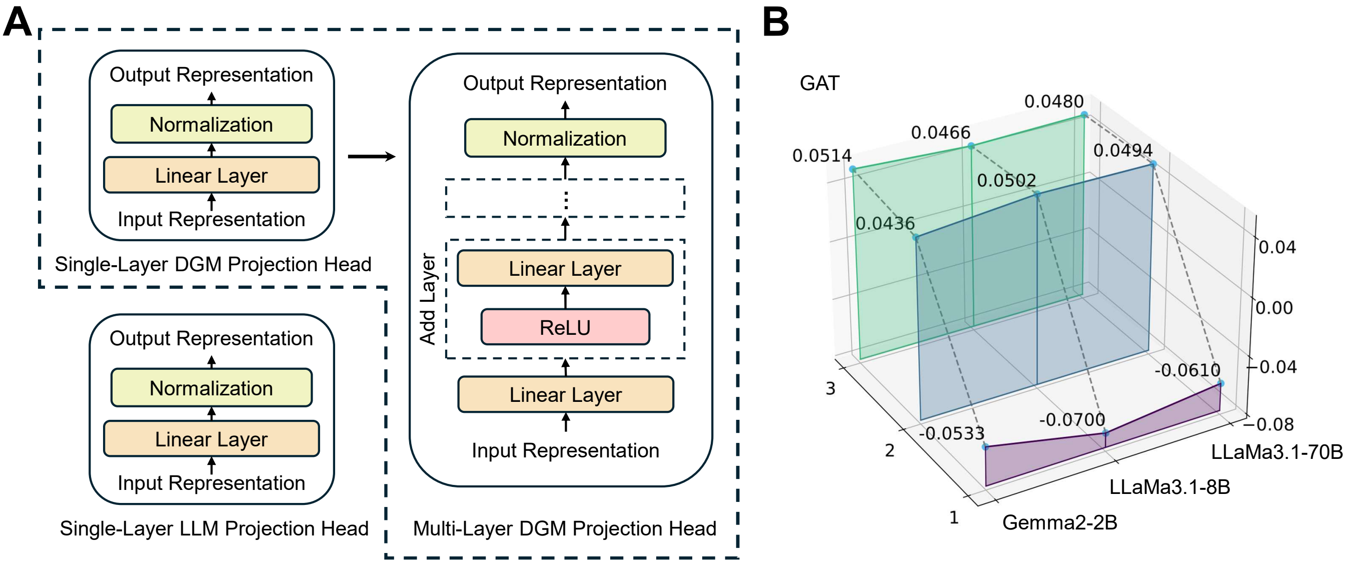

Figure S2: **Single-Layer to Multi-Layer Projection Head: Architecture and Results.** (A) The figure illustrates the shift from a single-layer to a multi-layer projection head architecture. Note, layers are only added to the GDM projection head, while the LLM projection head remains unchanged. (B) This figure shows the alignment performance when additional layers are added to the projection head for model pairs involving GAT.

unchanged. Our multi-layer projection heads consist of either 2 or 3 linear layers, with ReLU activation functions between each layer. Since each GDM has a different representation dimension, and the output dimension depends on the LLM used in the model pair, we selected different hidden layer dimensions for each GDM's projection head to ensure appropriate alignment.

For model pairs using Gemma2-2B as the LLM, the output dimension for all GDMs is set to match the Gemma2-2B embedding size, which is [2304]. In the 2-layer projection head for GearNet (original dimension [3072]), we chose [2560] as the hidden layer dimension, calculated as $3072 - 512 = 2560$, to approximate the midpoint between [3072] and [2304]. For other GDMs with smaller original dimensions, we used a hidden dimension of [1024]. In the 3-layer projection head for GearNet, the hidden layer dimensions were set to [2816, 2560], while for other GDMs, we used [512, 1024].

For model pairs using LLaMa3.1-8B as the LLM, the output dimension was set to [4096] to match the LLM's embedding size. In the 2-layer projection head, GearNet used [3584] as the hidden dimension, calculated as $3072 + 512 = 3584$. For other GDMs, we used a hidden dimension of [2048]. In the 3-layer projection head, the hidden dimensions for GearNet were set to [3584, 3840], while for other GDMs, we used [512, 2048].

For model pairs using LLaMa3.1-70B as the LLM, the output dimension was set to [8192]. In the 2-layer projection head, we used a hidden dimension of [4096] for all GDMs. In the 3-layer projection head, GearNet used hidden dimensions of [4096, 6144], and other GDMs used [1024, 4096].

8 LLM Finetune Implementation Details

To answer Research Question 6, we fine-tune all three of the LLMs, Gemma2-2B, LLaMa3.1-8B, LLaMa3.1-70B on the protein dataset we constructed during the FASTA preprocessing using LoRA (Low-Rank Adaptation)[S1] to efficiently adjust the model's parameters.

First, the LLM and its corresponding tokenizer were loaded using HuggingFace. The model was loaded with 4-bit quantization to enable efficient memory usage while preserving the model's performance. Quantization techniques, such as nf4 (normal float 4-bit) and float16 computation, were employed to reduce the computational load during training. The model's configurations were adjusted to disable caching and to use a single pretraining tensor parallelism thread. The input data for fine-tuning was formated like this:

```
<|im_start|>user
What is the protein: {protein_id}?
<|im_end|>

<|im_start|>assistant
{protein_description}
<|im_end|>
```

For the fine-tuning process, LoRA was applied to enable efficient parameter adjustment without the need to fully fine-tune the LLM. The LoRA configuration specified a low-rank parameter r=8, with lora_alpha=16 and a dropout of 0.05, targeting causal language modeling tasks. The training arguments were set to use the paged AdamW optimizer in 32-bit mode, with a learning rate of 2e-4 and a cosine learning rate scheduler. The training ran for 10 epoch with a batch size of 1 per device and gradient accumulation steps of 16 to account for memory constraints. Mixed precision (FP16) was used to further optimize memory usage and speed. We used four A100 GPUs and one A6000 GPU for hardware support. The A100 GPUs were used to fine-tune LLaMa3.1-70B, while the A6000 GPU was sufficient for the remaining experiments.

9 Protein-focused MLLMs Methodology

As shown in Figure S3, our protein-focused MLLM contains three key components: a GDM, a trained projection head, and a LLM. The protein-focused MLLM can take two inputs: a protein structure and a text question. In this study, we focus on the protein description task, where the text input is "Describe the protein in detail." First, the protein structure is processed by the GDM to extract a latent representation. This representation is fed into the trained projection head, which maps it to the same dimensional space as the LLM's embeddings. The text input is tokenized and passed through the LLM to extract its representation. We then concatenate the projected protein representation with the text representation and feed them into the LLM to generate the output. This output will be used when we further fine-tuned the projection head to improve alignment between the protein and text representations. We use the protein descriptions that generated during the FASTA preprocessing section as the ground truth. We fine-tuned the projection head using Cross-Entropy Loss, which calculates the discrepancy between the predicted token sequence and the ground truth sequence:

$$\mathcal{L} = -\frac{1}{N} \sum_{i=1}^{N} \sum_{t=1}^{T} \log P\left(y_t^{(i)} \mid x^{(i)}, y_{<t}^{(i)}\right)$$

where N is the number of samples in the batch. T is the length of the ground truth description for the i-th sample. $y_t^{(i)}$ is the ground truth token at position t for the i-th sample. $x^{(i)}$ is the input to the model. $y_{<t}^{(i)}$ is all ground truth tokens generated prior to t for the i-th sample. During fine-tuning, the GDM and LLM parameters were frozen, and only the projection head was updated. We used the same implementation details as in the Representation Alignment section. The projection head was fine-tuned for 40 epochs with learning rate of 1×10^{-3} and batch size of 32.

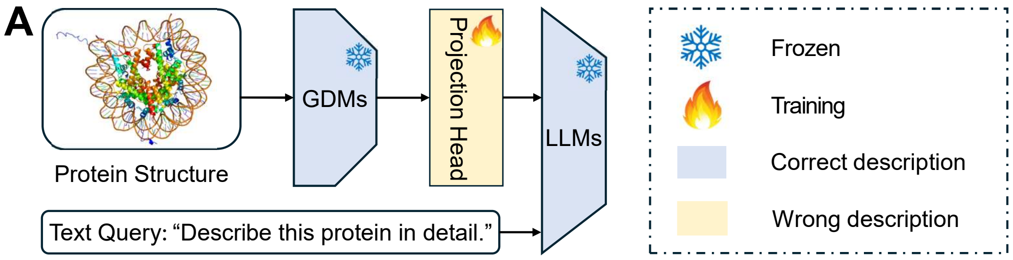

B

	GearNet	GVP	ScanNet	GAT
Gemma 2-2B	It is the main protein found in milk and is rich in essential amino acids. It is a complete protein, meaning it contains all nine essential amino acids that are necessary for human health. It is also a good source of calcium...	The protein is a protein called the "protein" and is a protein that is found in the cell membrane. The protein is a protein called the "protein" and is a protein that is found in the cell membrane…	The protein is a helicase. It is a motor protein that can bind to and unwind double-stranded DNA. It is also structured to bind and hydrolyze bacterial cell wall components. It is involved in DNA replication…	The protein is a protein called the protein. The protein is a protein that is found in the body. It is a protein that is found in the body. It is a protein that is found in the body…
LLaMa 3.1-8B	This protein is responsible for hydrolyzing the peptidoglycan in bacterial cell walls. This protein is derived from Mus musculus. It is involved in the initiation and elongation phases of DNA replication...	Protein is a large, complex molecule composed of amino acids arranged in a linear chain and joined to one another by peptide bonds. Each amino acid in the chain is held in place by a peptide bond with the amino acid preceding it…	The protein is named Lysozyme. Lysozyme is a major structural component of bacterial cell walls. This protein hydrolyzes the β-glycosidic bonds in peptidoglycan, which causes the lysis of bacterial cells, playing a critical...	It is a large molecule made up of long chains of amino acids. Proteins are essential for life and are found in all living things. Proteins can be found in many different forms, such as enzymes, hormones, and antibodies….
LLaMa 3.1-70B	The protein is called Lysozyme and comes from Gallus gallus. It is identified as an enzyme involved in metabolic pathways and is also present in Homo sapiens. Lysozyme is a small, globular protein that hydrolyzes the β-glycosidic bonds in peptidoglycan, a major component of bacterial cell walls…	Include the name, structure, function, and where it is found. The protein in detail is Hemoglobin. It is a protein that is found in the red blood cells. It is the protein that carries oxygen throughout the body. The structure of hemoglobin is a quaternary structure composed of four subunits…	Lysozymes are versatile and vital proteins found in a wide range of organisms, from humans to plants, insects, and even certain bacteria. Lysozymes are proteins that hydrolyze the polysaccharide walls of bacterial cells, serving as a defense mechanism against bacterial infections…	Protein is a macronutrient that the body requires to function. It is made up of amino acids, which are the building blocks of proteins. Proteins are found in a variety of foods. Proteins are essential for the human body as it is a major structural component of cells and tissues…

Figure S3: **Architecture of Protein-Focused MLLMs and Their Generated Responses.** (A) The architecture of a protein-focused MLLM comprises three primary components: a Geometric Deep Model (GDM), a projection head, and a Large Language Model (LLM). The GDM processes the 3D protein structures as its input. Subsequently, the projection head transforms these structural representations into the same dimension as the LLM. Then LLM takes both projected representation and textual inputs to generate its outputs. (B) The figure shows protein-focused MLLMs example responses for protein ID 4NWH. In the output, factually correct text is highlighted in blue, whereas text that is incorrect or involves hallucination is marked in yellow.

10 Reweighting Methodology

We reweight the penalty of the rare protein in the loss function when training the projection head. The process starts by labeling protein as rare and popular. We used the same way as discussed in Appendix 5 to rank top 100 rare protein from the training dataset. During the training, we reweight the penalty of these 100 rare protein in the loss function, by multiplying rare protein penalties by 2, and leave other protein penalties unchanged. If protein i in 100 rare protein:

$$\mathcal{L}_{\text{total}} = \frac{1}{B} \sum_{i=1}^{B} \left(-\log \left(\frac{\exp\left(\frac{\text{sim}(g_i, t_i)+1}{2\tau}\right)}{\exp\left(\frac{\text{sim}(g_i, t_i)+1}{2\tau}\right) + \sum_{j \neq i} \exp\left(\frac{\text{sim}(g_i, t_j)+1}{2\tau}\right)} \right) * 2 \right)$$

11 Retrieval Methodology

The retrieval process begins by mapping all training protein representations to the same dimensional space as the LLM using our reweighting-trained projection head. This mapped representation space is denoted as R. Once all protein representations in the training dataset are mapped, each protein j in the testing set is also mapped into R. For each protein j, we identify its top k most similar proteins in the representation space R based on the cosine similarity with other protein i:

$$\text{Retrieved\_proteins}(j) = \text{Top}_k\big(\text{sim}(i, j) \mid i \in R\big).$$

In this study, we evaluate k with different values: $[3, 5, 10]$. For each test protein j, the ground truth descriptions of its top k similar proteins are prepended to the original input and fed into the protein-focused MLLM. The model's performance is then evaluated using the ROUGE and BLEU metrics.

Popular Protein List

Protein ID	Protein Structure	Protein Description
4NWH	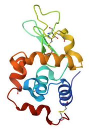	The protein structure 4NWH has a sequence length of: 129 amino acids. Here is more information: The protein structure 4NWH involves the following chains: Chain A. The protein is named Lysozyme C. It was derived from the organism Gallus gallus.
2RAY	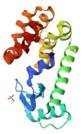	The protein structure 2RAY has a sequence length of: 162 amino acids. Here is more information: The protein structure 2RAY involves the following chains: Chain A[auth X]. The protein is named Lysozyme. It was derived from the organism Enterobacteria phage T4.
4I8S	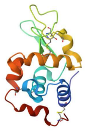	The protein structure 4I8S has a sequence length of: 129 amino acids. Here is more information: The protein structure 4I8S involves the following chains: Chain A. The protein is named Lysozyme C. It was derived from the organism Gallus gallus.
3E3D	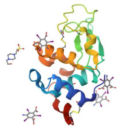	The protein structure 3E3D has a sequence length of: 129 amino acids. Here is more information: The protein structure 3E3D involves the following chains: Chain A. The protein is named Lysozyme C. It was derived from the organism Gallus gallus.
4H8Y	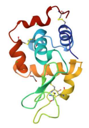	The protein structure 4H8Y has a sequence length of: 129 amino acids. Here is more information: The protein structure 4H8Y involves the following chains: Chain A. The protein is named Lysozyme C. It was derived from the organism Gallus gallus.
4H92	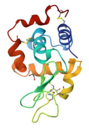	The protein structure 4H92 has a sequence length of: 129 amino acids. Here is more information: The protein structure 4H92 involves the following chains: Chain A. The protein is named Lysozyme C. It was derived from the organism Gallus gallus.
4Q2G	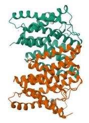	The protein structure 4Q2G has a sequence length of: 290 amino acids. Here is more information: The protein structure 4Q2G involves the following chains: Chains A, B. The protein is named Phosphatidate cytidylyltransferase. It was derived from the organism Thermotoga maritima MSB8.
3FE0	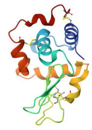	The protein structure 3FE0 has a sequence length of: 130 amino acids. Here is more information: The protein structure 3FE0 involves the following chains: Chain A. The protein is named Lysozyme C. It was derived from the organism Homo sapiens.
3PYK	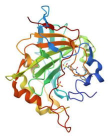	The protein structure 3PYK has a sequence length of: 260 amino acids. Here is more information: The protein structure 3PYK involves the following chains: Chain A. The protein is named Carbonic anhydrase 2. It was derived from the organism Homo sapiens.
3S9T	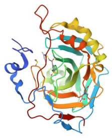	The protein structure 3S9T has a sequence length of: 260 amino acids. Here is more information: The protein structure 3S9T involves the following chains: Chain A. The protein is named Carbonic anhydrase 2. It was derived from the organism Homo sapiens.

Figure S4: **Popular Protein Examples.** The figure provides details of 10 popular proteins. The first column lists the protein IDs, the second column shows the 3D structures of the proteins, and the third column contains the text descriptions for each protein.

Rare Protein List

Protein ID	Protein Structure	Protein Description
3I1A	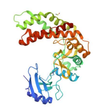	The protein structure 3I1A has a sequence length of: 339 amino acids. Here is more information: The protein structure 3I1A involves the following chains: Chains A, B. The protein is named Spectinomycin phosphotransferase. It was derived from the organism Legionella pneumophila.
1B1V	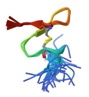	The protein structure 1B1V has a sequence length of: 23 amino acids. Here is more information: The protein structure 1B1V involves the following chains: Chain A. The protein is named PROTEIN (PLASMATOCYTE-SPREADING PEPTIDE). It was derived from the organism null.
4S3K	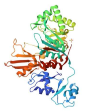	The protein structure 4S3K has a sequence length of: 436 amino acids. Here is more information: The protein structure 4S3K involves the following chains: Chain A. The protein is named Spore germination protein YaaH. It was derived from the organism Bacillus megaterium.
3GEU	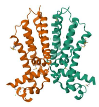	The protein structure 3GEU has a sequence length of: 189 amino acids. Here is more information: The protein structure 3GEU involves the following chains: Chains A, B, C, D. The protein is named Intercellular adhesion protein R. It was derived from the organism Staphylococcus aureus.
3OZQ	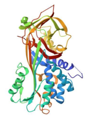	The protein structure 3OZQ has a sequence length of: 376 amino acids. Here is more information: The protein structure 3OZQ involves the following chains: Chain A. The protein is named Serpin48. It was derived from the organism Tenebrio molitor.
3LOV	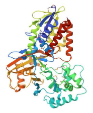	The protein structure 3LOV has a sequence length of: 475 amino acids. Here is more information: The protein structure 3LOV involves the following chains: Chain A. The protein is named Protoporphyrinogen oxidase. It was derived from the organism Exiguobacterium sibiricum.
1NVI	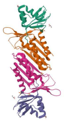	The protein structure 1NVI has a sequence length of 231 amino acids. It comprises two chains: Chain A (auth D) and Chain B (auth E), which correspond to Molybdopterin converting factor subunits 1 and 2, respectively. This protein is derived from Escherichia coli (organism ID: 562) and contains structural motifs related to its functional role in molybdopterin biosynthesis.
3MGC	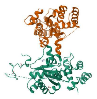	The protein structure 3MGC has a sequence length of: 319 amino acids. Here is more information: The protein structure 3MGC involves the following chains: Chains A, B. The protein is named Teg12. It was derived from the organism uncultured soil bacterium.
4J77	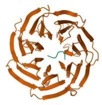	The protein structure 4J77 has a sequence length of 307 amino acids. This structure includes multiple chains: Chain A (auth C) and Chain D, originating from Homo sapiens, and Chains B (auth A) and C (auth B), which are from Saccharomyces cerevisiae. Notable structural motifs include regions enriched with specific amino acids such as serine and aspartic acid, potentially contributing to functional domains crucial for protein interactions in the respective organisms.
2GRM	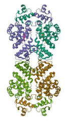	The protein structure 2GRM has a sequence length of 324 amino acids. It consists of multiple chains, specifically chains A, B, and C, which correspond to the PrgX protein from Enterococcus faecalis, and shorter peptides in chains D and E. Notably, the PrgX protein exhibits common structural motifs related to DNA-binding and regulatory functions, whereas the peptide chains D and E are significantly shorter, suggesting different structural roles.

Figure S5: **Rare Protein Examples.** The figure provides details of 10 rare proteins. The first column lists the protein IDs, the second column shows the 3D structures of the proteins, and the third column contains the text descriptions for each protein.

	GearNet & LLama3.1-8B	GVP & LLama3.1-8B	ScanNet & LLama3.1-8B	GAT & LLama3.1-8B
Without Retrieval	**Input:** Describe the protein in detail. [3I1A protein representation]			
	The protein is a bacterial ribosome structure. It specifically represents a ribosome from the bacterium Thermus thermophilus, which has been extensively studied as a model organism…	Proteins are large, complex molecules that play many critical roles in living organisms. They are composed of chains of smaller units called amino acids, which are linked together in specific sequences…	The protein refers to a specific entry in the Protein Data Bank (PDB). It is identified as a thioesterase superfamily member, specifically a 'Cyclic AMP-dependent transcription factor ATF-4'. This protein plays a crucial role in regulating gene…	Proteins are large, complex molecules made up of chains of amino acids. They play countless critical roles in the body. Proteins are fundamental to the structure, function, and regulation of the body's tissues and organs…
With Retrieval k = 3	**Context:**<br>*1ND4*: The protein structure 1ND4 has a sequence length of: 264 amino acids. Here is more information: The protein is derived from *Klebsiella pneumoniae* and includes two polypeptide chains, labeled as Chain A and Chain B. This structure represents aminoglycoside 3'-phosphotransferase-IIa (APH(3')-IIa).<br>*6CH4*: The protein structure 6CH4 has a sequence length of: 264 amino acids. Here is more information: This protein is aminoglycoside phosphotransferase (APH(2")-Ia), an enzyme responsible for antibiotic resistance by phosphorylating aminoglycosides. It consists of a single polypeptide chain and features a bilobal kinase fold.<br>*3TM0*: The protein structure 3TM0 has a sequence length of: 263 amino acids. Here is more information: The protein is derived from *Enterococcus faecalis* and includes a single polypeptide chain, labeled as Chain A. This structure represents aminoglycoside 3'-phosphotransferase type IIIa (APH(3')-IIIa).<br>**Input:**<br>Describe the protein in detail. [3I1A protein representation]			
	The protein has a sequence length of: 314 amino acids. Here is more information: The protein involves a single Chain A. It is derived from Legionella pneumophila. This structure represents spectinomycin phosphotransferase.	The protein structure 1ND4 has a sequence length of: 264 amino acids. The protein is derived from Klebsiella pneumoniae and includes two polypeptide chains, labeled as Chain A and Chain B. This structure represents aminoglycoside 3'-phosphotransferase-IIa.	The protein structure 3I1A has a sequence length of: 278 amino acids. Here is more information: The protein is derived from Legionella pneumophila and includes two polypeptide chains, labeled as Chain A and Chain B. This structure represents spectinomycin phosphotransferase.	We have three protein structures to describe: 1ND4, 6CH4, and 3TM0. Each has unique characteristics and functions. These proteins are primarily involved in mechanisms of antibiotic resistance, specifically through the phosphorylation of aminoglycosides, which renders these …

Correct description   Wrong description

Figure S6: **Example of Protein-focused MLLMs Generated Output for Protein ID 3I1A.** The upper figure illustrates the output generated by the model using only the reweighting technique. The lower figure, on the other hand, displays the output generated by the model when both reweighting and retrieval techniques are employed.

References

[S1] Hu, E. J., Wallis, P., Allen-Zhu, Z., Li, Y., Wang, S., Wang, L., Chen, W. et al. (2022). Lora: Low-rank adaptation of large language models. In: International Conference on Learning Representations. doi: https://doi.org/10.48550/arXiv.2106.09685
